# Influence of silica-fume’s addition technique and curing conditions on microstructure, physical and mechanical properties of geopolymer

**DOI:** 10.1038/s41598-025-23157-x

**Published:** 2025-11-04

**Authors:** Hala A. Hossein, A. A. El-Kheshen, M. F. Zawrah

**Affiliations:** 1 Refractories, Ceramics and Building Materials Department, National Research Centre, 12622 Dokki, Cairo, Egypt; 2 Glass Research Department, National Research Centre, 12622 Dokki, Cairo, Egypt; 3 Pharos University in Alexandra, Smoha, Alexandria, Egypt

**Keywords:** Geopolymer, Metakaolin, Mixing technique, Silica fume, Curing conditions, Properties, Chemistry, Materials science

## Abstract

Geopolymer as ecofriendly and energy saving material is estimated as excellent building material in construction sector. The current study focuses on studying the influence of silica fume mixing technique, its contents (2.3, 4.6, 6.9%) and curing conditions on microstructure, physical and mechanical properties of metakaolin-based geopolymers. Two mixing methods and two various curing conditions (air and 100% relative humidity) were utilized for production of geopolymers. The prepared geopolymers were investigated by various techniques such as x-ray diffraction (XRD), Fourier transforms Infrared spectroscopy (FTIR) and Scanning electron microscope (SEM). The bulk density, apparent porosity and compressive strength were also examined. The results revealed that the aforementioned parameters were significantly affected the properties of the produced geopolymers. The physico-mechanical properties were improved when the geopolymers prepared by the proposed method (Dissolving silica fume in 12 M NaOH before mixing with metakaolin), as compared to that prepared by other method (mixing of silica fume with metakaolin before addition of NaOH). The properties of geopolymers cured in air were better than that cured in 100% relative humidity. The optimum percentage of silica fume that gave the best properties was 2.3%, after which the properties were reduced. The geopolymers that contained 2.3% silica fume, exhibited the highest compressive strength (45.13 and 39.97 MPa), while the geopolymers that contained 6.9% silica fume displayed the lowest compressive strength (17.11 and 15.5 MPa), when cured in air or 100% RH, respectively.

## Introduction

 The growing of world population emphasizes the necessity for sustainable development of industry, urbanization and energy saving. It is well known the cement production is the 2nd biggest product that liberates carbon dioxide (CO_2_) in the atmosphere. Every ton of cement production is emitting about 0.87 tons of CO_2_, i.e. 5–8% of worldwide CO_2_ emission, in addition to other SO_3_ and NOx polluting oxides. This causes pollution of the environment, global warming, climate change problems and acid rains^1–6^. Moreover, this industry is creating a huge quantity of by-product waste that also pollutes the environment and can also consume large amounts of natural raw materials (clay, sand and limestone). Furthermore, the production of cement consumes a large quantity of energy when compared with the production of geopolymer that save energy. Recently, a new type of binding material that can replace the cement has paid a great attention; this category of materials called geopolymers. In addition to their excellent properties and technological applications, they can be fabricated from natural raw materials and/or by recycling of a lot numbers of industrial solid wastes^7,8^. During the advancement of sciences and development of technology with the necessity to produce clean products and save the energy, the scientists are stimulated to endorse new materials like geopolymers. Geopolymers are 3D amorphous alumino-silicate binding materials fabricated from natural or waste sources of alumina and silica, in presence of alkali or acid activators. The natural materials include clays like metakaolin while the solid wastes include waste-clay, fly-ash, slags, rice husk-ash, silica fume, etc. This means that inorganic geopolymerization process is occurred without releasing pollutant-gases and with low energy consumption^9–14^. During geopolymerization process, the tetrahedral chains of SiO_4_ and AlO_4_ are formed and connected together by participation of the oxygen atoms as a substitute^15^. Recently, the geopolymers are applied as alternatives to ordinary Portland cement (OPC) for binding the concretes, as restoring materials, acid and fire resistant materials, decoration, removal of pollutants from wastewater, etc. Several studies have been reported on the integration of natural pozzolans^16–19^, industrial and agricultural waste products in construction of geopolymer materials. These wastes include granulated blast furnace slag (GBFS)^20–22^, silica fume (SF)^11,23–25^, rice husk ash^26–28^, fly ash^1,28,29^, red mud^30,31^, silico-manganese (Si–Mn) slag, Cu–Ni slag and lead slag^32–34^ and Feeders’ and Cyclones’ waste-clays^35^. Both metakaolin and silica fume exhibit strong pozzolanic characteristics that support the formation and strength development of geopolymers. Their ability to react with alkaline activators and calcium hydroxide contributes to a denser, more robust microstructure, resulting in improved mechanical properties and durability of geopolymer-based materials. As such, these materials are essential for optimizing the performance of geopolymers in various applications.

The choice of alkali activators is crucial for geopolymer formation, including alkali hydroxides, alkali silicates, carbonates, aluminates, and sulfates. They are utilized alone or mixed according to the precursor necessities^36,37^. It has been reported in literatures that the type and/or concentration of activator, the fineness of raw material and form of alumino-silicate precursor play an important role in the activation rate of geopolymerization. The appropriate concentration of NaOH activator significantly affects the dissolution process of the starting materials and the formation of a structure with compacted solid particles, which in turn enhances the mechanical properties of the resulting geopolymers^38–40^. The activation by concentrated NaOH is recommended to dissolute of alumino-silicate solids owing to its higher ability to free the silicate and aluminate monomers. Moreover, the existence of sodium silicate (pre-added or in-situ formed) with concentrated sodium hydroxide has also a significant effect on the geopolymer properties^41–43^. Thus, in the present study, in-situ formed sodium silicate is formed during the proposed processing technique through the reaction between NaOH and silica fume.

Kaolin is one of the natural abundant mineral in the earth’s crust and belongs to kaolinite clay group that comprises of one tetrahedral-sheet and one-octahedral sheet in its structure. Kaolin is converted into metakaolin (MK; amorphous pozzolanic active alumio-silicate) after heat treatment in the range of 600–850 °C^44^. The activation of MK with concentrated NaOH solution and sodium silicate plays a significant role in geopolymerization process; where the finesse of MK and the presence of concentrated Na_2_O with soluble silicate have a considerable impact on compactness of geopolymer matrix^45–48^. Silica fume is a by-product produced after production of silicon and ferrosilicon alloy by reduction of high purity quartz with coal in an electric furnace; it has been utilized in different applications^49–53^. It is a very fine and amorphous pozzolanic active material with more that 95% SiO_2_ content. It exhibits good solubility in concentrated NaOH at low temperature and form excellent alkali activator (composed of sodium silicate and excess sodium hydroxide) suitable for geopolymer fabrication. Different study reported that the silica fume has been utilized as exporter of interacting SiO_2_ to form water glass alkali activator^54,55^. Moreover, SF can replace sodium silicate alkali activator to generate MK-based geopolymers by directly mixing with MK followed by interacting with NaOH to obtain SF-MK based geopolymers^56,57^. When silica fume (SF) is mixed directly with MK, the silicon metal in SF has a negative effect on the structure of prepared geopolymers because of the existed silicon metal can react with alkali and form hydrogen gas that leads to porous structure formation. Generally, the curing conditions (temperature, atmosphere & relative humidity) as well as the molar ratio of SiO_2_/Al_2_O_3_, Na_2_O/Al_2_O_3_ and SiO_2_/NaO are the key fundamentals for improving the properties of geopolymers^58^.

The main purposes of this work are to study the effect of mixing technique (i.e. by dissolving of SF in concentrated sodium hydroxide before addition of metakaolin or by mixing solid SF with metakaolin then mixing with concentered sodium hydroxide) and curing conditions (air or relative humidity) on phase composition, physical properties, microstructural changes and physico-mechanical properties of MK-based geopolymers. Several suitable techniques were utilized to inspect the phase composition, porosity, bulk density microstructure, and mechanical properties of fabricated geopolymers.

The significances of this research are: (i) Production of clean product (geopolymer) with saving energy, (ii) proposing of a suitable processing technique for production of geopolymer with improved properties, and (iii) the prepared geopolymers are suitable for different construction applications.

## Materials and experimental methods

### Materials

In this study, the starting raw material employed for production of geopolymer was Egyptian kaolin. It was supplied by general company for ceramic & porcelain products (Sheeni), Egypt. The raw kaolin was converted into metakaolin (active amorphous aluminum silicate) by thermal treatment at 850 °C in a lab furnace. On the other hand, silica fume (SF – 94% SiO_2_) by-product was provided by Ferrosilicon Company in Edfo, Upper Egypt. Sodium hydroxide pellets (99%) were provided by Sigma Aldrich.

### Preparation of geopolymers by different mixing techniques and curing conditions

In the present study, the geopolymers were prepared by different mixing techniques and curing conditions. Two different techniques were utilized during mixing the alkali activator (sodium hydroxide) and silica fume with metakaolin. The first method includes directly mixing of silica fume with 12 M NaOH to synthesizing in-situ formed alkali activator that contains a mixture of sodium silicate, excess sodium hydroxide and excess unreacted silica fume. This activator was prepared by the reaction between silica fume powder and highly concentrated sodium hydroxide (12 M). This prepared alkali activator was then mixed with metakaolin to obtain geopolymer pastes. While the second method dealt with mixing of silica fume powder directly with metakaolin powder, then the concentrated sodium hydroxide solution (12 M) was added to the mixed powders to obtain geopolymer pastes. In all case, the geopolymers obtained by both methods were subjected for two curing conditions; (i) cured in air for 28 days or (ii) cured in 100% relatively humidity for 28 days. In the first method; typically, NaOH pellet (99% purity), was employed to prepare 12 M NaOH solution by dissolving in a tap water. Afterward, the solution was left in ambient condition until its high temperature is totally dropped down to maintain equilibrium and to avoid the quickening of geopolymer setting time. After preparation of concentrated sodium hydroxide solution, different percentages of silica fume (i.e. 2.3, 4.6 and 6.9 w/v %) per 100 ml of concentrated NaOH were added to prepare three alkali activators^11,20,24,34^. The mixtures were stirred using magnetic stirrer working at 200 rpm and room temperature. The mixing process was continued for about 30 min in order to prevent agglomeration of ultrafine silica fume powder. Then, the beakers-containing mixtures were closed. To obtain a significantly dissolved silica fume in alkali solution and formation of alternative alkali activators, the reaction process was continued for 8 h. The obtained activators included mixes of sodium silicate, excess sodium hydroxide and excess unreacted silica fume. These three solutions were utilized for production of three metakaolin-based geopolymers (i.e. A, B and C samples)^59–62^. The metakaolin and prepared alternative activator were mixed together for 10 min in a mixer to obtain uniform and workable geopolymer pastes. These pastes were quickly casted into a steel mold (25 × 25 × 25 mm), then positioned on the vibrating table and vibrated for 5 min to increase the compactness and to release any residual air bubbles. To increase the geopolymerization process, the prepared pastes were cured tranquil and activated at 60 °C for 24 h in a lab oven. Following to this step, the specimens were de-molded cured in air at ambient temperature or in 100% relative humidity (RH) for 28-days. To stop the curing process (hydration of geopolymer), the crushed geopolymer specimens were then subjected to drying at 105 °C for 24 h. The dried crushed samples were kept in closed holder until testing. For comparison, forth batch (D) was prepared by another technique; namely, the geopolymer was prepared by directly mixing of metakaolin and silica fume powders (sieved by 75 μm sieve), by porcelain ball mill for 30 min to obtain a completely homogenized powder. Afterward, concentrated NaOH activator (12 M) was mixed with the prepared powder mixture for 15 min in a mixer until getting a workable geopolymer paste. The fabricated geopolymer (D) was activated and cured in air or 100% RH by the same aforementioned conditions of the batches A, B, and C. Table 1 illustrates the batches composition and mixing techniques of prepared geopolymers.

**Table 1:** Batch composition and mixing technique of geopolymer pastes.

Batch name	*SF, g	NaOH (12 M), ml	SF/NaOH, (w/v %)	**MK, g	Mixing technique
A	1	43.3	2.3	100	(SF + NaOH) + MK
B	2	43.3	4.6	100	(SF + NaOH) + MK
C	3	43.6	6.9	100	(SF + NaOH) + MK
D	2	43.5	4.6	100	(SF + MK) + NaOH

* SF: Silica fume ** MK: Metakaolin

### Characterization of raw materials and prepared geopolymers

#### Chemical and phase compositions

The chemical composition of both raw kaolin and silica fume was investigated by Axios wavelength dispersion X-ray fluorescence (WD-XRF) sequential spectrometer, Panalytical, the Netherlands, 2009. The mineralogical phase composition of both raw materials and fabricated geopolymers was examined by x-ray diffraction technique using XꞌPert PRO PW3040/60 (PANalytical) diffractometer worked at 40 kV and 30 mA and equipped with monochromatic Cu-K*a* radiation source. The obtained data was recognized using XꞌPert high score software connected with PDF-2 database. The surface area of metakaolin and silica fume was determined by BET method Furthermore, the functional groups and compositions of prepared geopolymer were investigated by FTIR spectroscopy using JASCO FT/IR-6100. The spectra were recorded in the range of 400–4000 cm^−1^ with a resolution of 4 cm^−1^, at 25 °C.

#### Apparent porosity and bulk density measurements

To determine the apparent porosity^54^ and bulk density of prepared geopolymer specimens, the weight (wt.) of cubic-shaped samples was estimated using a four digital balance. On the other hand, the bulk volume (vb) was determined by measuring their dimensions using a digital caliper of 0.01 mm precision. The bulk density (ρb, in g/cm^3^) was then calculated according to the following equation:

i $$\rho b{\rm{ }} = {\rm{ }}wt./vb$$

ii $$\emptyset = {\rm{ }}100{\rm{ }} \times {\rm{ }}(vb - vg)/vb$$

The grain volume (vg) was consequently measured by using a Helium Quantachrome Pycnometer at 19 psi and room temperature. Afterward, porosity (Ø) was obtained using the following equation:

In order to obtain reliable values with higher accuracy, three geopolymer samples were utilized for measurements and the average values were calculated^63^.

#### Microstructure of prepared geopolymers

The microstructure and qualitative elemental analysis of prepared geopolymer were investigated by field emission scanning electron microscope, operating at 15 kV (FE-SEM, model FEJ Quanta 250, Netherlands), and coupled with an energy dispersive X-ray analyzer (EDX) unit.

#### Compressive strength (CS) of prepared geopolymers

The compressive strength of prepared geopolymers cured in air or 100% RH was tested by automatic hydraulic testing instrument type SHIMADZU with ultimate capacity of 1000 kN and at rate of 0.025 kN/mm^2^/s according to with EN 1015-11^64^ and EN 12390-6^65^. The recorded value was average of three readings.

## Results and discussion

### Characteristics of raw materials

The chemical analyses of silica fume and metakaolin dried at 105 °C as analyzed by X- ray fluorescence are illustrated in Table 2. It is indicated that metakaolin is mainly constitutes of SiO_2_ (59%), and Al_2_O_3_ (37%) in addition to some other minor oxides like Fe_2_O_3_, CaO, MgO, Na_2_O, K_2_O, and SO_3_. On the other side, silica fume is mainly composed of SiO_2_ (94%) with some other oxides as Fe_2_O_3_, CaO, MgO, Na_2_O, K_2_O, and SO_3_. The surface area of metakaolin and silica fume was 15 and 35.20 m²/g, respectively.

**Table 2:** Chemical composition of metakaolin and silica fume in mass-%.

Oxide/Wt%-	SiO_2_	Al_2_O_3_	Fe_2_O_3_	CaO	MgO	Na_2_O	K_2_O	SO_3_	LOI
Metakaolin	59.02	37.1	2.53	0.59	0.28	0.11	0.09	0.34	0.30
Silica fume	94.08	0.79	2.55	0.48	1.12	0.04	0.01	0.10	1.25

**Fig. 1:**
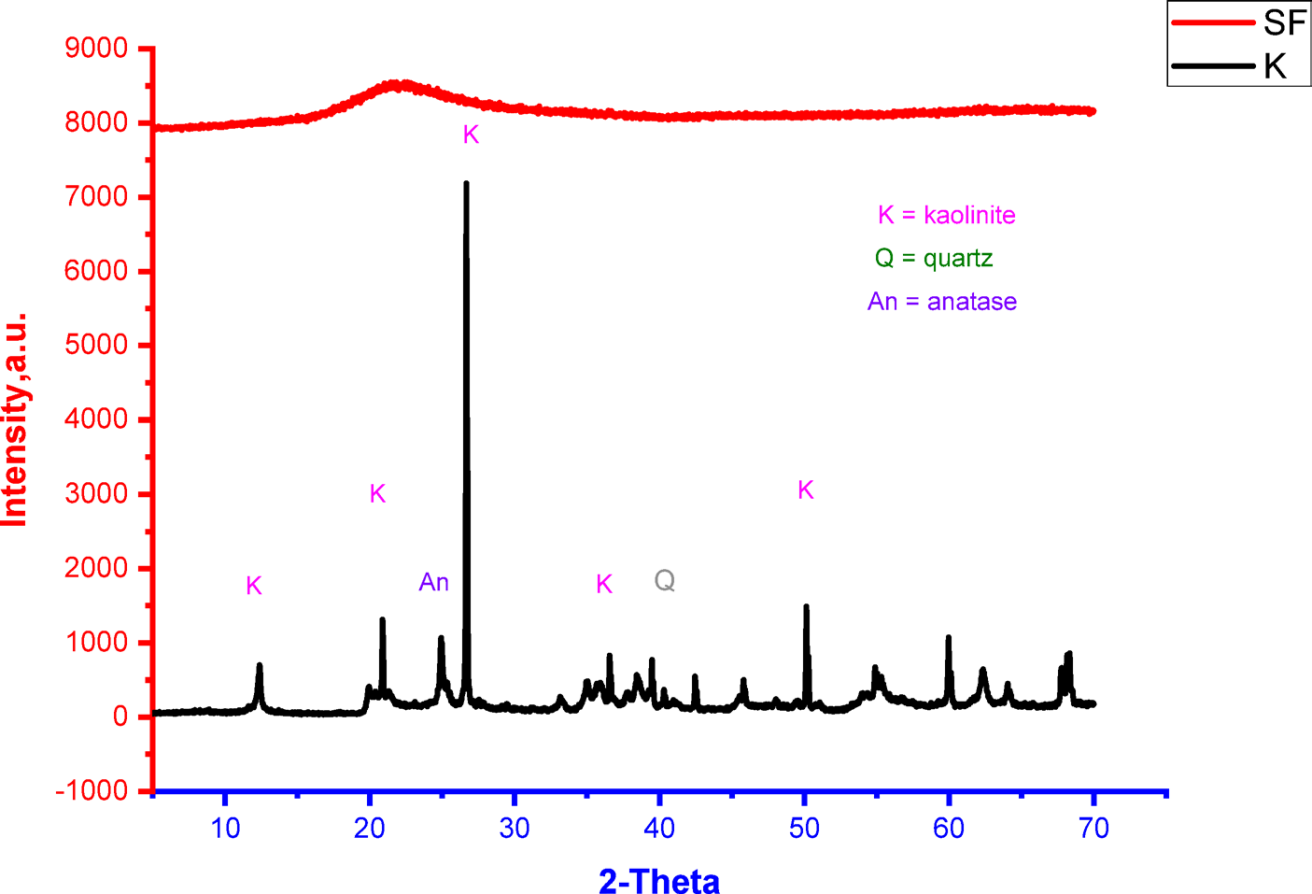
XRD patterns of metakaolin and silica fume waste.

Figure 1 shows XRD patterns of metakaolin (MK) and silica fume (SF) raw materials. It can be seen from the patterns that the silica fume is totally amorphous material as indicated from the broad hump between 2θ◦ = 15–40. On the other side, the metakaolin structure is intermediate between semi-crystalline and amorphous material. The peaks of kaolinite mineral, quartz in addition to a few amount of anatase are appeared in the pattern.

### Properties of fabricated geopolymers

#### Phase composition of prepared geopolymers

**Fig. 2:**
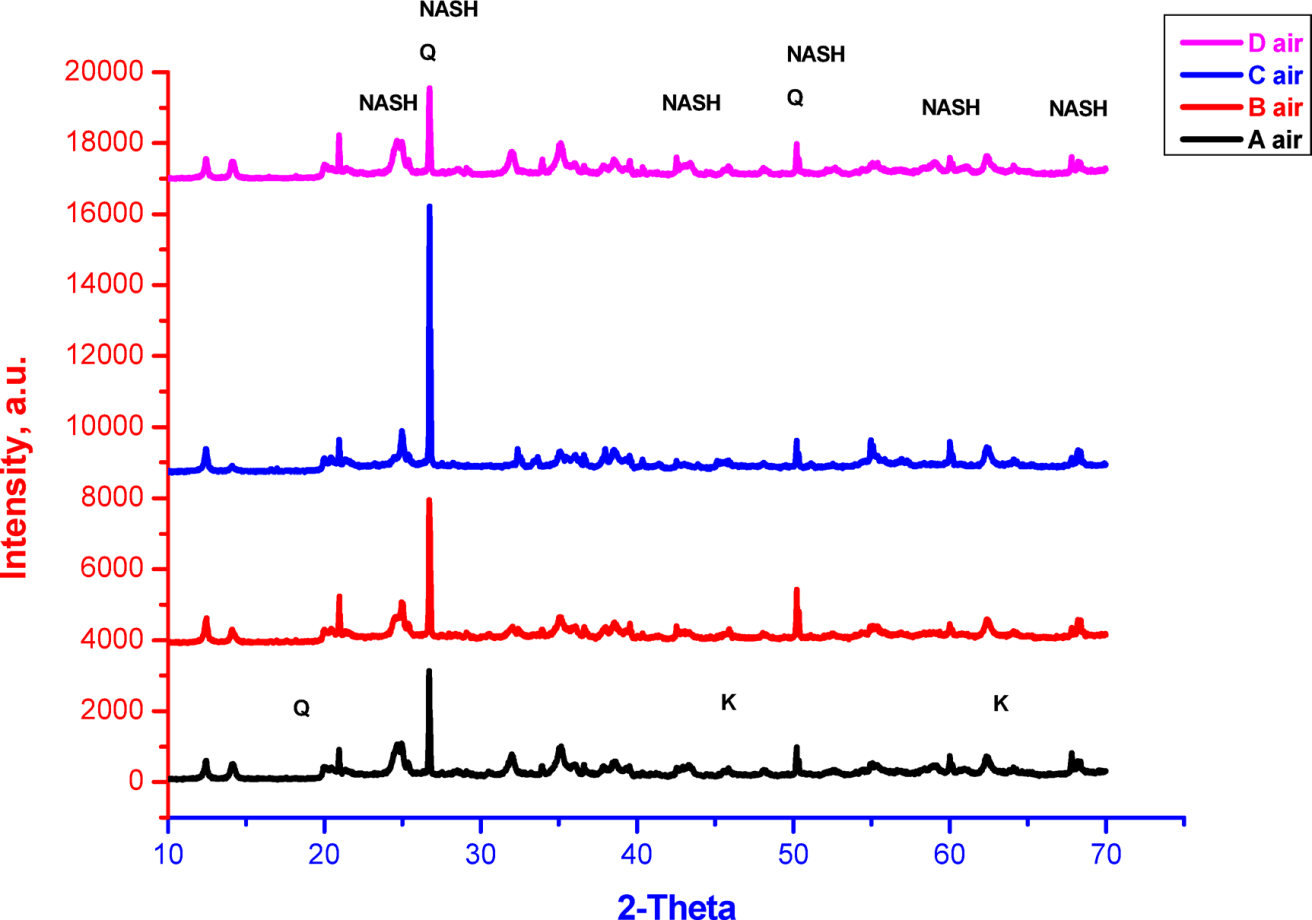
XRD patterns of geopolymers prepared by different techniques, cured in air, and having different silica fume contents.

**Fig. 3:**
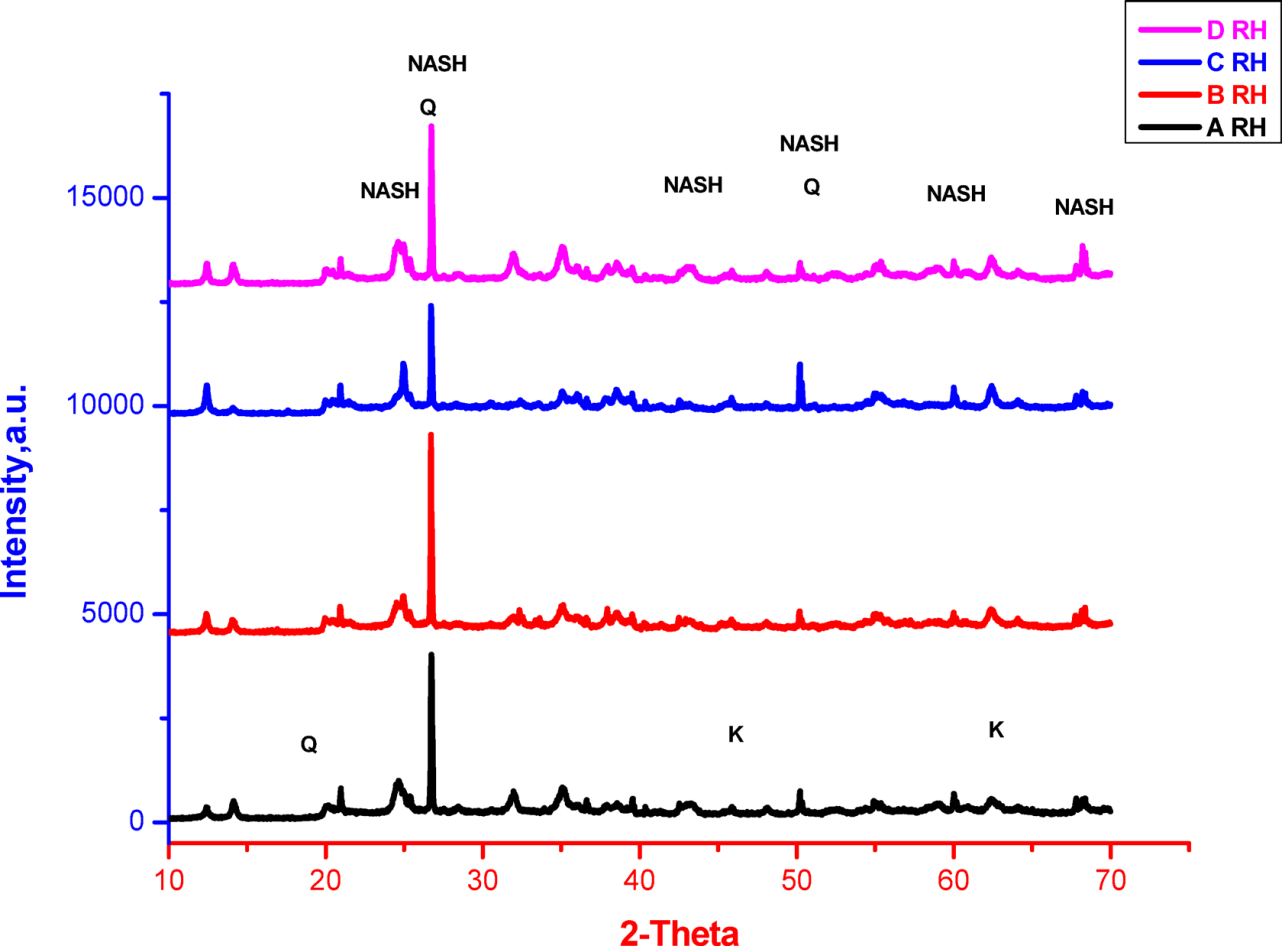
XRD patterns of geopolymers cured in 100% RH, prepared by different techniques and having different silica fume contents.

XRD patterns of geopolymers containing various ratios of silica fume, prepared using different mixing techniques and cured in either air or 100% relative humidity, are shown in Figs. 2 and 3, respectively. It is indicated from the figures that most of patterns are relatively similar with some deviations according to the silica fume content, mixing technique and curing conditions. All samples display the following phases with different percentages according to the aforementioned parameters; these phases are semi-crystalline or amorphous sodium aluminum silicate hydrate (NASH), quartz (Q) and minor amount of unreacted kaolinite (K) according to PDF files No. 00–005-0490, 01–073-5301 and 00–001-0527, respectively. The main peaks of NASH, quartz, kaolinite are detected at 2-theta equal to 24.56, 26.64 and 62.26, respectively. The formation of amorphous or semi-crystalline NASH phase indicates the geopolymerization of reactants with the formation of geopolymer networks^11,66–69^. On the other hand, the appearance of quartz and kaolinite phases indicates the incompletion of reaction or they are already existed with higher percentages than that needed for reaction to form geopolymer. As shown in Fig. 2, which displays the XRD patterns of geopolymers cured in air, sample A (containing 2.3% silica fume) exhibits lower peak intensity of quartz and greater broadness of NASH peaks, indicating a higher polymerization process compared to the other geopolymer samples. The increasing of quartz peak intensity and broadness reduction of NASH peaks as we going from the geopolymer A to C indicate the reduction of polymerization in that trend. For the geopolymer D that prepared by another technique (mixing of solid SF with metakaolin then mixing with NaOH liquid), the pattern exhibits lower peak intensity of quartz and higher broadness of NASH peaks, i.e. this sample gives relatively similar polymerization like the sample B. For the geopolymers cured in 100% relative humidity (Fig. 3), the peaks’ intensity of quartz and broadness of NASH peaks are relatively similar with small deviation as compared to that cured in air. This indicates that the relatively higher geopolymerization of the samples cured in air than the samples cured in 100% relative humidity. This can be confirmed by comparing the patterns of same sample cured in air or 100% relative humidity, that display the differences of peaks’ intensities of quartz and NASH, i.e. the amount of quartz and NASH in comparison with each other. It is worth to mention that the geopolymer prepared from only metakaolin without addition of silica fume has been reported elsewhere^14^. It exhibited only amorphous NASH phase with apparent porosity, bulk density and compressive strength values equal to 31%, 1.27 g/cm^3^ and 31 MPa, respectively. Furthermore, it has been also reported that the addition of solid silica fume together with solid metakaolin before adding the alkali activator led to deterioration of microstructure of prepared geopolymer with increasing the porosity due to the evaporation of hydrogen gas as a result of reaction between the silicon metal existed in silica fume with sodium hydroxide^11^. Thus, in the current research, the preparation technique is changed (as conducted in mixes A, B, and C), i.e. silica fume was added firstly to sodium hydroxide solution to get rid of hydrogen gas and to prepare a mixture of sodium hydroxide, sodium silicate and insoluble silica fume. The presence of soluble sodium silicate for some extent can improve the geopolymerization process and formation of more amorphous Si-O-Al gels. Moreover, the absence of hydrogen gas can reduce the porosity, improve the microstructure and consequently improve the mechanical properties. Furthermore, the existed insoluble silica fume for some extent can contribute in packing of geopolymer through filling some open pores and consequently improve the geopolymer properties. However, the addition of higher percentage of silica fume than 2.3%, leads to reduction of geopolymerization process due the existence of higher content of insoluble silica fume that might affect oppositely on the geopolymer formation specially when exceed the stoichiometry of free alumina needed for geopolymer formation^70^. Therefore, the suggested technique of preparation influences effectively on the formed phases, geopolymerization process and consequently on other properties. The improved properties of prepared geopolymers are also influenced by curing conditions that effect clearly on the geopolymerization process. This means that the findings of current study depended on three parameters, namely, preparation techniques, curing conditions and silica fume contents. The curing conditions (air or RH) are playing serious roles on the geopolymerization process especially with low or high content of silica fume. Generally, the presence of lower amount of silica fume with higher concentrated sodium hydroxide tends to form sodium carbonate crystals during the geopolymerization process that might consume large amount of sodium ions^71,72^. Also, the carbonation of geopolymers can occur through different processes, mainly influenced by environmental conditions like air-drying or relative humidity (RH). In case of carbonation by air-drying, this process typically involves exposure to CO₂ in the air, leading to the formation of carbonates. While some carbonation can enhance compressive strength in the short term, excessive carbonation may lead to micro-cracking and reduced long-term durability. In case of carbonation by high RH, it can be more detrimental. The presence of moisture can facilitate the reaction of CO₂ with the alkaline components in the geopolymer. This process may lead to a more uniform carbonation but can also result in a loss of mechanical strength due to the breakdown of the polymer matrix and increased porosity^73–75^. In all cases, the geopolymers cured in air exhibit better geopolymerization with slower carbonation than that cured in relative humidity. The mechanisms of carbonation in air and RH can be explained as follows:

##### Carbonation in air-dried geopolymers

1. Direct CO₂ absorption: When exposed to air, geopolymers can absorb CO₂ directly from the atmosphere. The reaction leads to the formation of carbonates.
2. Formation of carbonic acid: CO₂ reacts with water present in the geopolymer matrix, forming carbonic acid (H₂CO₃), which can further react with available alkaline components.
3. pH modification: The reaction with carbonic acid can lower the pH of the geopolymer, facilitating further reactions that lead to the formation of calcium and sodium carbonates.
4. Microstructural changes: Carbonation can lead to changes in the microstructure, potentially increasing porosity and affecting mechanical properties.

##### Carbonation in high relative humidity (RH) environments

1. Enhanced CO₂ solubility: In high RH conditions, the amount of water in the geopolymer increases, which enhances the solubility of CO₂, leading to a more pronounced reaction.
2. Carbonic acid formation: Similar to air-dried conditions, CO₂ reacts with water to form carbonic acid, but the higher water content may promote greater diffusion and reaction rates.
3. Hydration reactions: In high humidity, ongoing hydration reactions may occur alongside carbonation, influencing the overall chemistry of the geopolymer and potentially leading to the formation of more stable carbonate phases.
4. Increased reaction rates: The presence of more water can facilitate faster diffusion of CO₂ into the geopolymer matrix, resulting in more extensive carbonation and potentially different mineralogical products.

In summary, carbonation mechanisms in geopolymers involve reactions with CO₂ and water, leading to the formation of carbonates. The drying conditions significantly influence the kinetics and extent of these reactions, with air-dried samples typically showing slower carbonation rates compared to those dried in high RH environments.

#### Fourier transforms infrared spectrums (FTIR) of prepared geopolymers

**Fig. 4:**
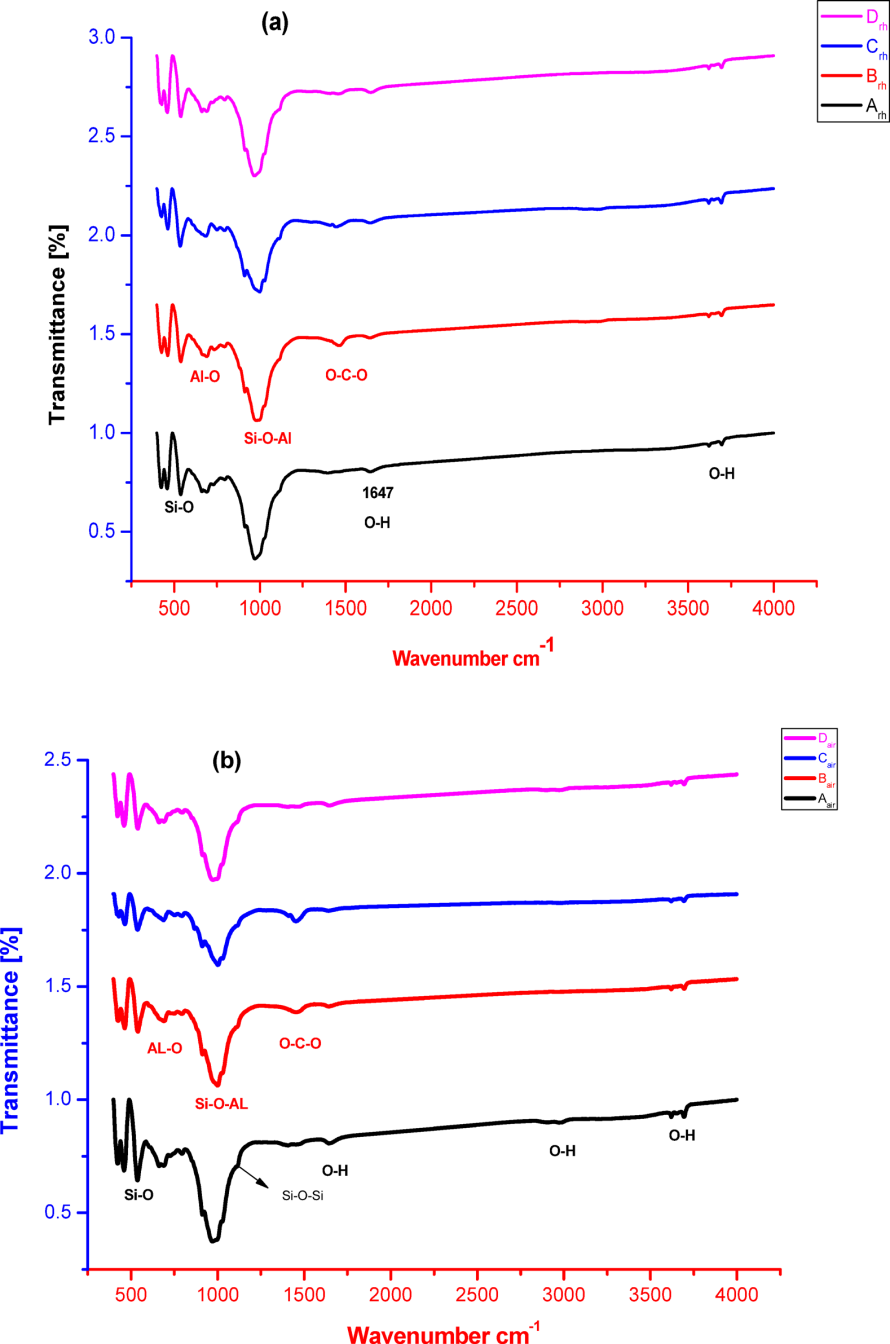
FTIR patterns of geopolymers cured in 100% RH (**a**) or in in air (**b**), prepared by different techniques and having different silica fume contents.

FTIR spectra of geopolymers A, B, C and D cured in RH and air for 28 days are shown in Fig. 4. The two bands appeared at 3705 and 3625 cm^−1^ are attributed to OH stretching vibration of water content in the prepared geopolymers. Furthermore, the band located in the range 1640–1660 cm^−1^ is assigned to OH-bending vibration. The band detected at about 1400 cm^−1^ demonstrates the carbonate group. Generally, the carbonation is relatively higher in the geopolymers A, B and C than the sample D. This carbonation might occur during the preparation of alkali activator mixes. The bands assigned in the range 822–1250 cm^−1^ (centered at 972 cm^−1^) is the main band of geopolymer i.e. Si-O-Al/Si-O-Si asymmetrical stretching vibrations (NASH gels)^76,77^. As indicated from the intensities of the aforementioned band in all prepared geopolymers, this means that the increasing of silica fume in the batches reduces the geopolymerization process due to the presence of excess unreacted silica fume more than the needed for geopolymerization. This consequently affects the other properties^78^. When comparing the intensity of main band related to Si-O-Al centered at 972 cm^−1^ in the geopolymers A or D cured in air and RH, it can be seen that the band intensity is increased in sample A cured in air than that cured in RH confirming the higher geopolymerization in geopolymer A cured in air. On contrary, in the geopolymer D, the Si-O-Al band intensity is increased in RH than in air. This means that the RH is effective and increased the geopolymerization when the geopolymer is prepared by the second supposed method. The band at 667 cm^−1^ is assigned for Al-O while the band at 534 cm^−1^ is allocated for O-Si-O bond of silica.

**Fig. 5:**
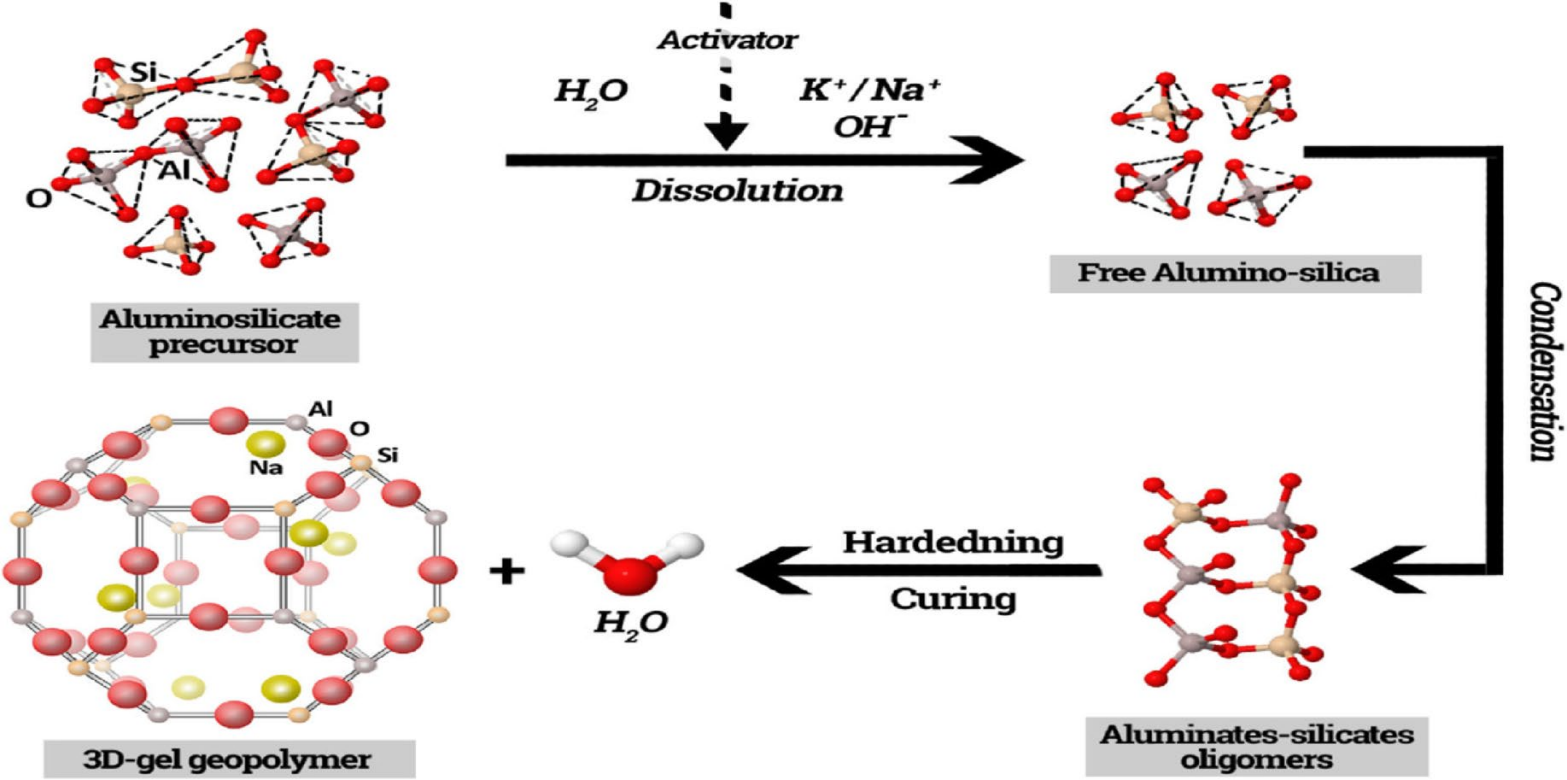
Stages of geopolymerization process.

It is well known that the mechanism of geopolymerization is happened in two steps^40–43^. The first stage contains the dissolving of metakaolin by alkali activator with the formation of alumino-silicate oligomers. While the second stage includes poly-condensation and bonding of oligomers to for the geopolymer networks as shown in Fig. 5. These bonds are poly-sialate (–Si–O–Al–O–), or poly-sialate-siloxo (Si–O–Al–O–Si–O) or poly-sialate-disiloxo (Si–O–Al–O–Si–O–Si–O–).

#### Physical properties of prepared geopolymer

**Fig. 6:**
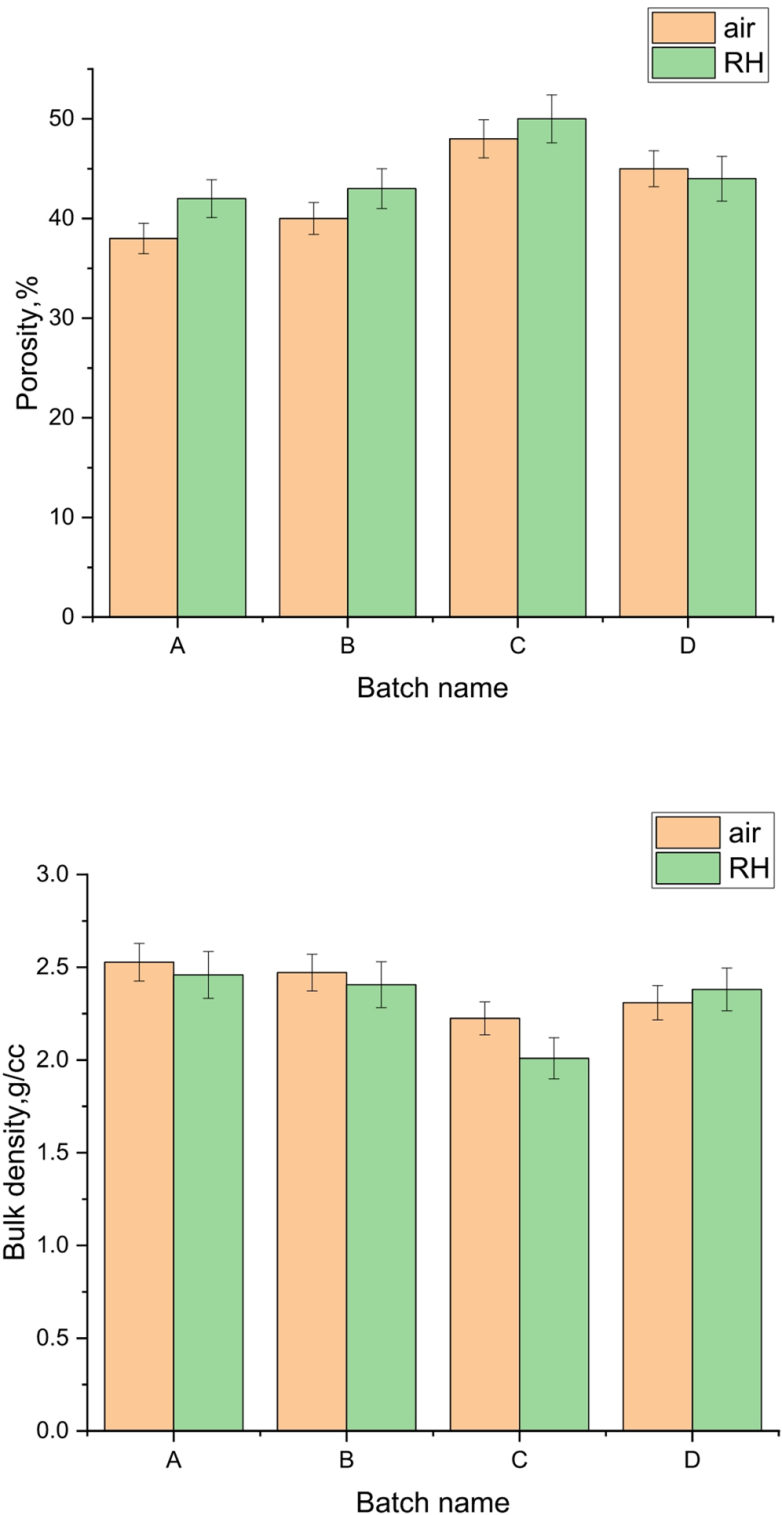
Apparent porosity (**a**) and bulk density (**b**) of geopolymers prepared by different techniques and cured in air or 100% RH.

The apparent porosity and bulk density of prepared geopolymers A, B, C and D cured in air or 100% RH are displayed Fig. 6. It is well known that the apparent porosity and bulk density have an efficacious impact on the properties of solid material specially geopolymer^35,43^. It can be seen form the figure that the apparent porosity increases with increasing the silica fume content as we going from the sample A to C, and then relatively decreases again in the geopolymer D. Also, the porosity of the geopolymers cured in air is lower than that cured in RH. The obtained apparent porosities for the geopolymers cured in air are 38, 39.5, 47.5 and 45% while that cured in RH are equal to 42.3, 44.2, 47.5 and 43%, respectively. This trend confirms the reduction of geopolymerization with increasing the amount of added silica fume due to the excess unreacted silica in the geopolymer network. This means that the optimum amount of silica fume needed to give higher geopolymerization by the suggested technique is 2.3%; after which the geopolymerization is decreased. Increasing the amount of geopolymer network in the structure leads to reducing the porosity^79,80^. The results of physical properties support the XRD results. The bulk density goes in the opposite trend to the apparent porosity one. The values of the bulk density for geopolymers A, B, C, and D cured in air are equal to 2.53, 2.50, 2.3 and 2.4 g/cm^3^, while that for geopolymers cured in RH are 2.46, 2.40, 2.15 and 2.3, respectively.

#### Microstructure of prepared geopolymers

**Fig. 7:**
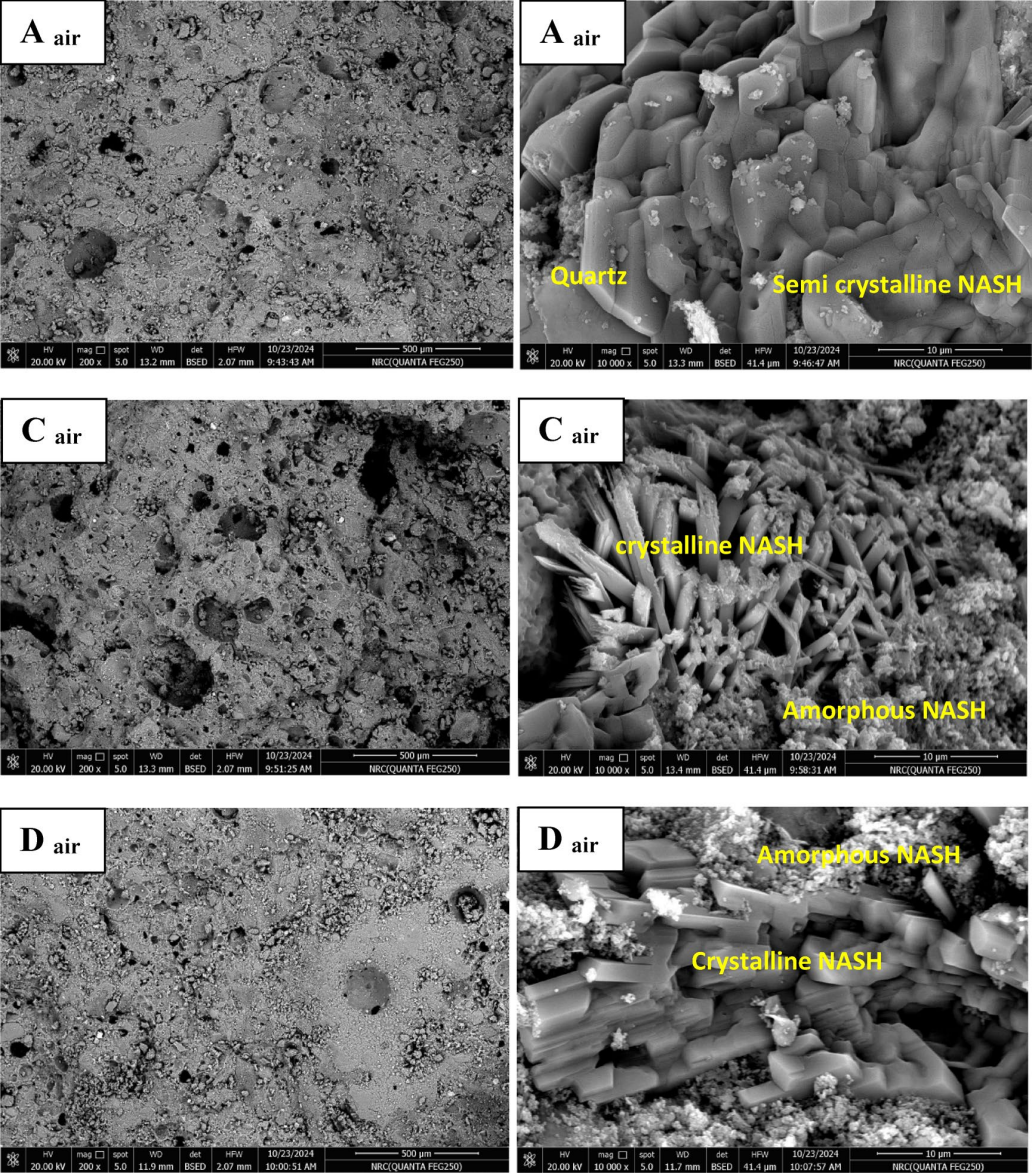
SEM images (different magnifications) of geopolymers A, C and D cured in air.

**Fig. 8:**
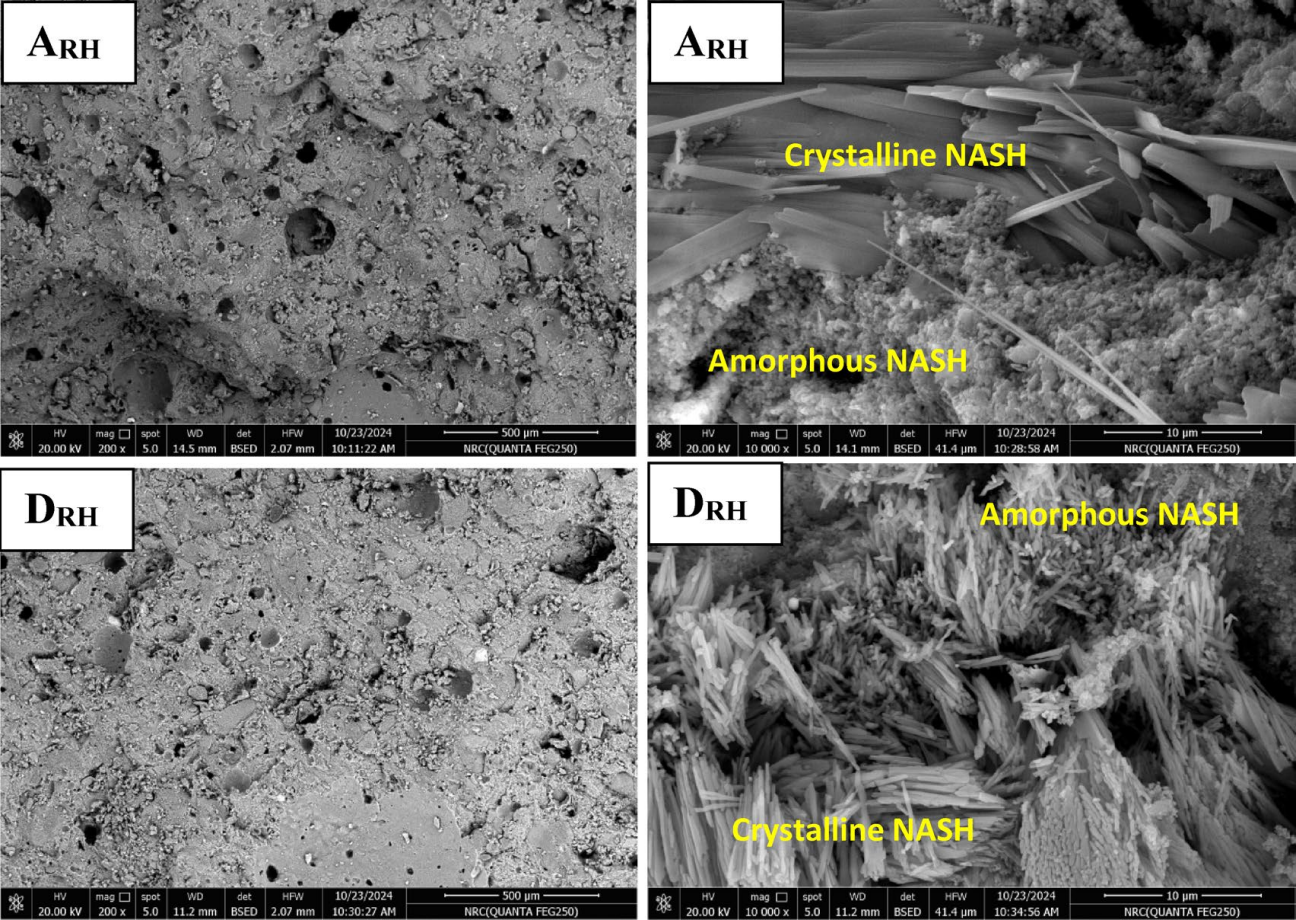
SEM images (different magnifications) of geopolymers A and D cured in RH.

**Fig. 9:**
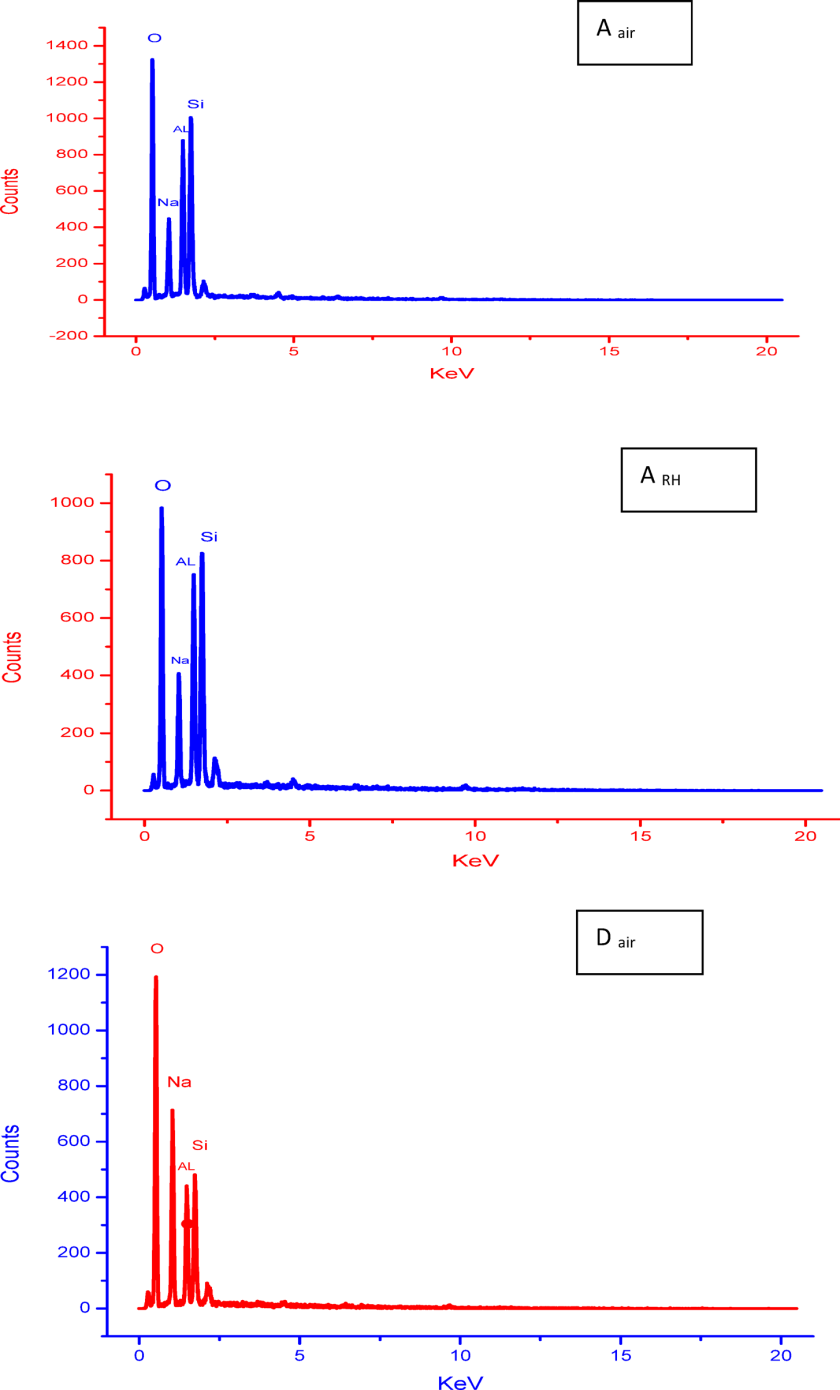

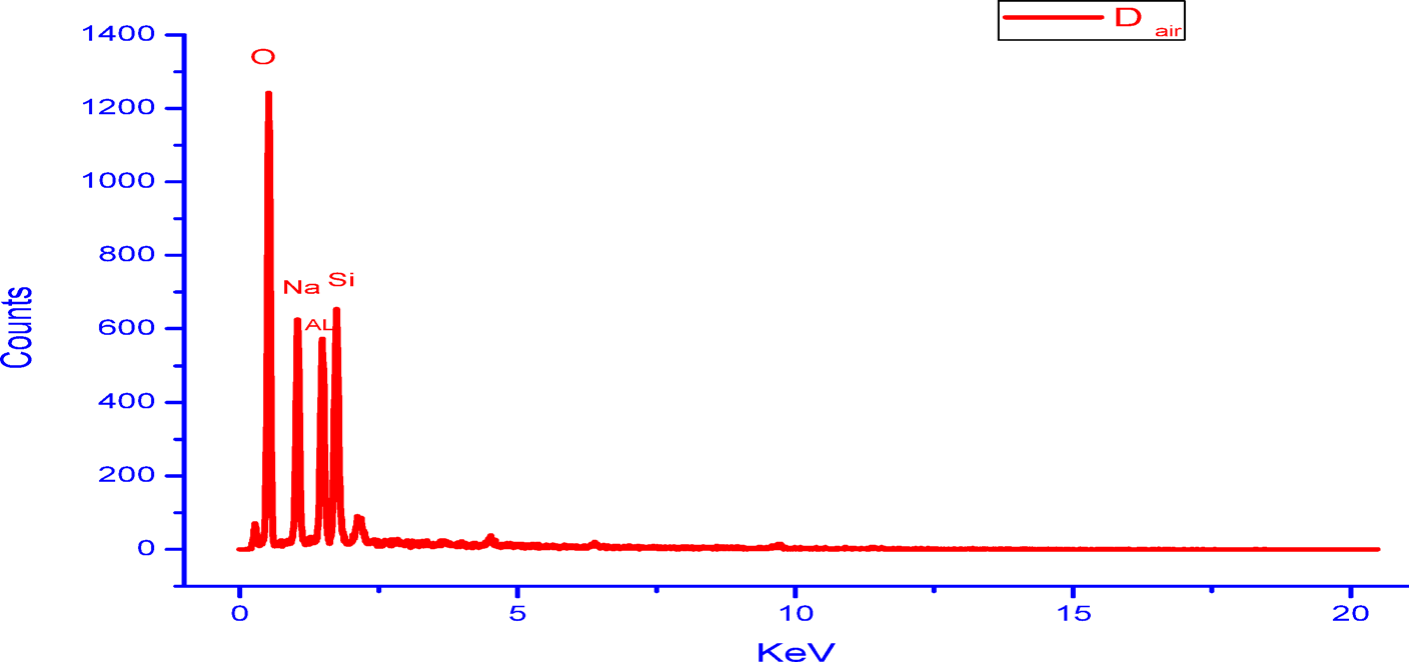
EDX analyses of geopolymers A and D cured in air or 100% RH for 28 days.

It is well known that the microstructure of geopolymer is influenced by different parameters like degree of geopolymerization and formation of amorphous or semi-crystalline NASH network gel, amount of unreacted reactants, porosity and new formed phases. Figure 7 shows SEM images (different magnifications) of geopolymers A, C and D cured in air for 28 days. As seen in the microstructures, the most important noticed textural or morphological features in the microstructure are the compactness and homogeneity. They are too high in case of geopolymer A followed by geopolymer D then it is reduced in the geopolymer C that exhibits porous structure. This is due to the higher degree of geopolymerization in geopolymer A than the other geopolymers. These features are exactly appeared in the low magnification images. The second important feature detected in the microstructure is the formation of amorphous gel and semi-crystalline NASH network structures. Its amorphous amounts are higher in the geopolymer A than the other C and D geopolymers. A few amounts of unreacted aluminosilicate and quartz are detected in the geopolymer structure; especially in the geopolymer C. With increasing the amount of added silica fume, the formed semi-crystalline silicate phases tend to be longer in length. This feature is obviously appeared in the higher magnification SEM images. Figure 8 shows SEM images (different magnifications) of geopolymers A and D cured for 28 days in 100% relative humidity. In comparison with the samples cured in air, the microstructure of geopolymers cured in RH exhibit relatively higher porosity with increasing the amount and lengths of formed long semi-crystalline silicate phases. It’s important to note that for geopolymers A, B, and C, the concentrated sodium hydroxide partially dissolves silica fume into soluble silicate, releasing active silica and creating a highly reactive alkali activator. This alkali activator reacts with alumina and silica in metakaolin forming alumino-silicate gel in the microstructure. This gel forms the matrix of the microstructure and interconnected together without micro cracks formation. This phenomenon is optimum in case of geopolymer A while it reduces in geopolymer B and C. These samples (B and C) exhibits more pores dispersed in the gel matrix and these pores produce from evaporation of water moisture that form air bubbles within the gel structure^81^. Unreacted metakaolin particles increase also the porosity of the structure; it can be coated with thin layer of geopolymers that prevent its further activation. Also, the geopolymers cured air is better than that cured in RH. Figure 9 displays EDX analysis of geopolymers A and D cured in air and RH, respectively. These results confirm the results of XRD and microstructure. As indicated from the higher peak intensity of aluminum and silicon in geopolymer A, this means that the quantity of alumino-silicate geopolymer is higher in case of geopolymer A than the geopolymer D. It can concluded that the nature of starting materials, type of alkali activator, technique of fabrication and degree of geopolymerization have intensive effect on the microstructure of formed geopolymers^9,11,13,14^.

#### Compressive strength of prepared geopolymers

**Fig. 10:**
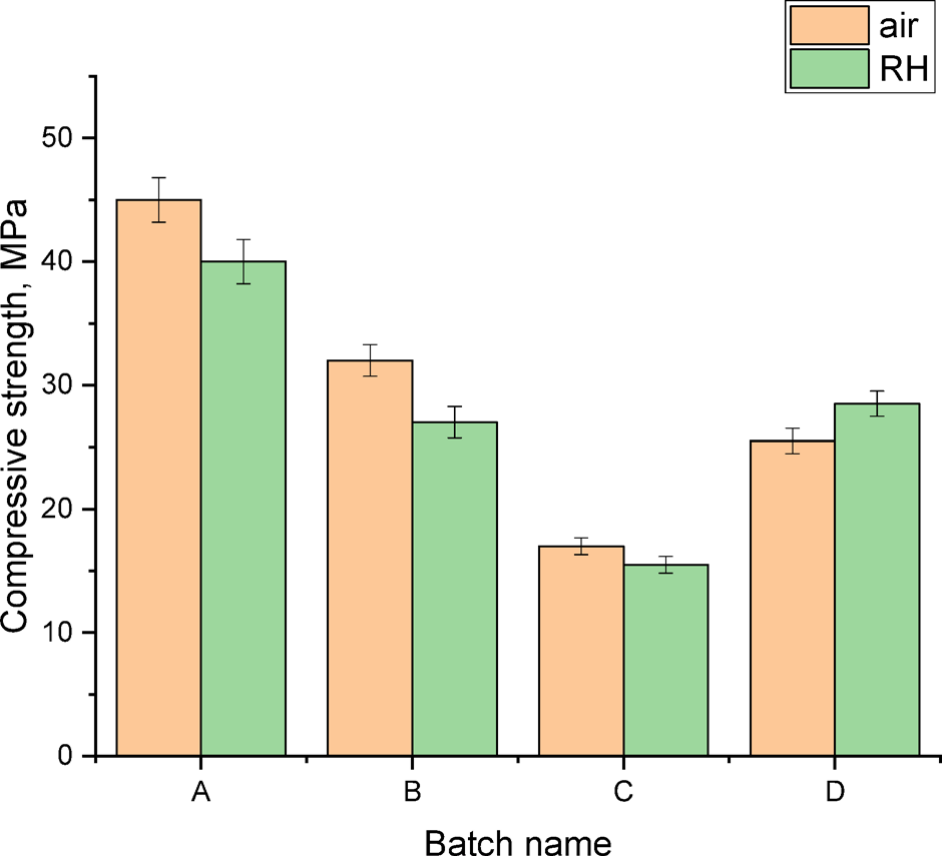
Compressive strength of prepared geopolymers cured in air or 100% RH for 28-days.

Figure 10 shows compressive strength (CS) values of geopolymers A, B, C and D cured for 28 days in air or 100% relative humidity (RH). As mentioned before, the compressive strength of geopolymer fabricated from metakaolin without addition of silica fume was 31 MPa^14^. As indicated in Fig. 10, the addition of silica fume and the selection of fabrication technique have a positive effect on the compressive strength. In comparison with geopolymer fabricated from metakaolin, the geopolymer (A) that included 2.3% silica fume, displays higher compressive strength (about 45 MPa) among the other geopolymers samples. This due to the higher degree of geopolymerization produced from the presence of suitable amount of formed sodium silicate and silica fume which improve the geopolymerization and packing of the microstructure networks. Moreover, with further increasing the amount of added silica fume, i.e. 4.6 and 6.9%, the compressive strength is reduced due to the reduction of geopolymerization degree. The existence of higher percentages of silica fume, more than the needed for geopolymer formation, leads to increasing the porosity and deterioration of network structure. Furthermore, the presence of higher amount of silica in the system causes the reduction of alumina index and decreases the solution alkalinity which in turn depresses the dissolution of kaolin particles. The geopolymer D (prepared by mixing SF solid with MK then adding NaOH) exhibits lower compressive strength than the sample that has same composition but prepared by other technique (prepared by mixing SF solid with NaOH liquid then adding MK) specially that cured in air. On the other hand, the geopolymer D cured in RH displays higher compressive strength than that cured in air. In this case, the system still needs some water to complete the geopolymerization; it acquires them for the humidity thus it gives higher compressive strength. However, all geopolymers A, B and C (prepared by mixing SF solid with NaOH liquid then adding MK) cured in air exhibits better compressive strength than that cure in air. The partially dissolved silica fume in highly concentrated sodium hydroxide (12 M), leads to forming soluble sodium silicate that participates for some extent in the geopolymerization reaction and then enhances the compressive strength. The presence of soluble silicate and sodium hydroxide, with aluminosilicate reactants, leads to generation of polymeric Si-O-Al-O bonds that forms amorphous alumino-silicate gel known as sodium alumino-silicate hydrate (NASH) as confirmed by XRD)^82–90^. In conclusion, the geopolymer A exhibits the highest CS (45.13 and 39.97 MPa), while the geopolymer C displays the lowest compressive strength (17.11 and 15.5 MPa) when cured for 28 days in air or 100% RH, respectively. Also, the geopolymers A, B and D are considered as suitable binding materials that consume lower energy with lower emission of harmful gases when compared with cement production.

## Conclusion

The following remarks can be concluded:

- Metakaolin-based geopolymer properties have been successfully improved by varying mixing technique, silica fume content and curing conditions (air or 100% relative humidity).
- Dissolving silica fume in concentrated sodium hydroxide (12 M) before mixing with metakaolin (first proposed mixing technique), had a positive effect on the properties of geopolymers compared to that prepared by other mixing technique (mixing SF solid with MK then adding NaOH).
- The optimum percentage of silica fume that gave the best properties was 2.3%, after which the properties were reduced. The addition of higher amount of silica fume led to increasing the amount of unreacted silica and reducing alumina index which consequently influenced on the amount of formed geopolymer.
- In case of direct mixing of silica fume to metakaolin, the existed silicon metal in SF led to evaporation of hydrogen gas due to the reaction with NaOH. This gas increases the porosity. But in the first proposed mixing method, we can get rid of hydrogen gas during the preparation of alkali activator mix with the formation of soluble sodium silicate that can promote the geopolymerization process.
- Regarding the curing conditions, the geopolymers cured in air were better than cured in water due to the lower carbonation of sample cured in air than that cured in water. The lower carbonation closes the pores and prevents more carbonation while higher carbonation leads to increasing the pores and deterioration of structure. The geopolymer A exhibited the highest CS (45.13 and 39.97 MPa), while the geopolymer C displayed the lowest compressive strength (17.11 and 15.5 MPa) when cured for 28 days in air or 100% RH, respectively.

## Data Availability

“All data generated or analyzed during this study are included in this published article.”

## Abbreviations

CS: Compressive strength
EDX: Energy dispersive X-ray
FTIR: Fourier transforms infrared spectroscopy
GBFS: Granulated blast furnace slag
K: Kaolin
kV: Kilo volt
MPa: Mega pascal
MK: Meta kaolin
OPC: Ordinary portland cement
Q: Quartz
M: Molar concentration
RH: Relative humidity
SEM: Scanning electron microscope
SF: Silica fume
Si-Mn: Silico-manganese slag
NASH: Sodium aluminum silicate hydrate
XRD: X-ray diffraction
XRF: X-ray fluorescence
3D: Three dimension
